# Medical Items

**Published:** 1895-09

**Authors:** 


### *MEDICAL ITEMS.*

The Governor has appointed Dr. O. B. Bush, of Mayhaw, Principal Physician to the Penitentiary, in place of Dr. Thos. M. McIntosh, resigned.

We have received from the A. L. Koursh Company, 921 Milwaukee avenue, Chicago, specimen copy of an excellent and complete "Case Recorder," for use in general medicine and gynecology by physicians and hospitals. It is well gotten up by Dr. S. B. Lyon, of Chicago, and will be found convenient and well adapted for the careful recording of cases.

The ninth annual meeting of the American Orthopedic Association will be held in Chicago, September 17, 18 and 19, in the Columbus Memorial Building, 103 State street. On the same dates the American Dermatological Association will meet at the Windsor Hotel, Montreal, Canada. President, Dr. S. Sherwell, New York; Secretary, Dr. C. W. Allen, 640 Madison avenue, New York.

The seventh annual meeting of the Tri-State Medical Society of Alabama, Georgia, and Tennessee will be held at Chattanooga on Tuesday, Wednesday, and Thursday, October 8th, 9th, and 10th. A large attendance is expected. An attractive program will be announced later. Papers have been promised by J. B. Murfree, J. B. Cowan, J. E. Woodbridge, R. P. Johnson, W. E. B. Davis, Richard Douglas, R. M. Cunningham, and others.

Here is the latest combination of science with practical utility. We learn that "Professor Esmarch has invented suspenders made of elastic webbing, which can be quickly detached from the trousers and converted into an Esmarch bandage," and we are advised that lumbermen, train-hands, policemen, engineers, etc., will do well to use the Esmarch suspender. The next thing will be a combination collar-button, which may be used in emergencies for intestinal anastomosis.

---

The latest invention of the serum therapists is *antivenene*. Dr. Thos. R. Fraser, of Edinburgh, has injected the venom of the rattlesnake and cobra, in increasing doses, into rabbits and cats, and he found that they could finally receive enough of the poison to kill a horse. The blood-serum (antivenene) of these animals thus rendered immune to ordinary doses of the serpent venom was used in protective inoculations upon other animals. Dr. Fraser expects this method to be made applicable to man, and that it will be speedily introduced into India, where the annual death-rate from snake-bite is considerable. Dr. Fraser's publication appeared in the *British Medical Journal*, June 15, 1895.

---

In a late case, *California Fig Syrup Company vs. Frederick Stearns & Company*, Detroit, in which the former sought to restrain the latter from using the words "fig syrup" on one of its preparations, it developed on trial that "Syrup of Figs" (California Co.) is "nine-twentieths syrup of figs, ten-twentieths fluid extract of senna, and one twentieth a mixture of rochelle salts, aromatics, and water." Witness for the complainant testified that one thousand gallons of the syrup would not contain more than one or two gallons of the active juice of the fig. For this and other good reasons Judge Swan threw the case out of court, saying that the law "will not lend its aid to foster this delusion of the public or countenance such deceit." This seems to be a very proper judge.

